# FOXK1 facilitates cell proliferation through regulating the expression of p21, and promotes metastasis in ovarian cancer

**DOI:** 10.18632/oncotarget.19713

**Published:** 2017-07-31

**Authors:** Li Li, Miao Gong, Yu Zhao, Xiujun Zhao, Quanhai Li

**Affiliations:** ^1^ Department of Histology and Embryology, Hebei Medical University, Hebei, China; ^2^ Department of Immunology, Hebei Medical University, Hebei, China

**Keywords:** FOXK1, proliferation, p21, metastasis, ovarian cancer

## Abstract

Ovarian cancer is one of the most common cancer in the world. FOX family plays essential function in multiple cancers. In our work, FOXK1 was found to up-regulate in ovarian cancer tissue samples and cell lines; moreover, the expression of FOXK1 was correlated with tumor size, metastasis and poorly prognosis. To evaluate the function of FOXK1 in ovarian cancer, we performed colony formation analysis, CCK-8 assay and cell cycle analysis to determine the effect of FOXK1 on cell proliferation and cell cycle. We found that FOXK1 obviously improved the ability of cell proliferation through promoting cell cycle. Furthermore, ChIP assay and luciferase reporter assay indicated that FOXK1 facilitated cell cycle through regulating the expression of p21, but FOXK1 had no effect on cell apoptosis. In addition, wound healing assay and transwell invasion analysis demonstrated that FOXK1 promoted migration and invasion in ovarian cancer. In conclusion, our work indicate FOXK1 plays a key function in the ovarian cancer, it promotes cell proliferation and metastasis. FOXK1 serves as a novel molecular therapy target in ovarian cancer.

## INTRODUCTION

Ovarian cancer is one of the most common cancer in the world [1]. During the development of new therapies and treatments, the five-year survival rate of patients had been increased. However, because patients were always diagnosed at an advanced-stage, ovarian cancer still had a high mortality rate [2]. Understanding the detail mechanism of ovarian cancer is helpful for us to find the therapy target.

Forkhead box (FOX) family has been found to play important function in several cell process, including embryonic development [3] and organogenesis [4]. Moreover, there are many reports indicate that FOX family regulates physiological processes [3, 5], including cell cycle [6], cell signaling [7] and metabolic processes [8]. Therefore, abnormal expression of FOX family protein results in cancer development [9-11]. Forkhead box k1 (FOXK1) belongs to FOX family, as a transcription factor, it recognizes and binds DNA consensus sequence, WRTAAAAYA, to regulate transcription [12, 13]. Previous studies indicate that FOXK1 regulates c-myc, p21 and cdc2 gene in mice [14]. Moreover, there are also many reports demonstrate that FOXK1 takes part in tumorigenesis [15, 16]. In addition, FOXK1 inhibition suppresses cell proliferation in human osteosarcoma cancer cells [17]. However, the role of FOXK1 in ovarian cancer is still unknown.

Cell proliferation is a complex cell program, it is regulated by numerous cell process, including cell cycle, cell apoptosis and so on. p21, also known as p21^WAF1/CIP1^, is mediated by p53. p21 plays a key function in G_1_ growth arrest [18, 19]. p21 regulates cell cycle arrest in response to multiple stimuli and there by promoting DNA repair.

Here, our work demonstrates that FOXK1 is significantly high expression in ovarian cancer tissues and cell lines. Moreover, high expression of FOXK1 predicts poor prognosis in ovarian cancer patients. In addition, FOXK1 promotes the proliferation through transcriptionally regulating *p21.* FOXK1 also facilitates metastasis in ovarian cancer cells. Our work firstly find the effect of FOXK1 on cell proliferation and metastasis in the ovarian cancer, and explain the molecular mechanism of FOXK1 in cell proliferation.

## RESULTS

### FOXK1 is up-regulated in ovarian cancer and the expression of FOXK1 is corrected with poor prognosis of ovarian cancer

**Figure 1:**
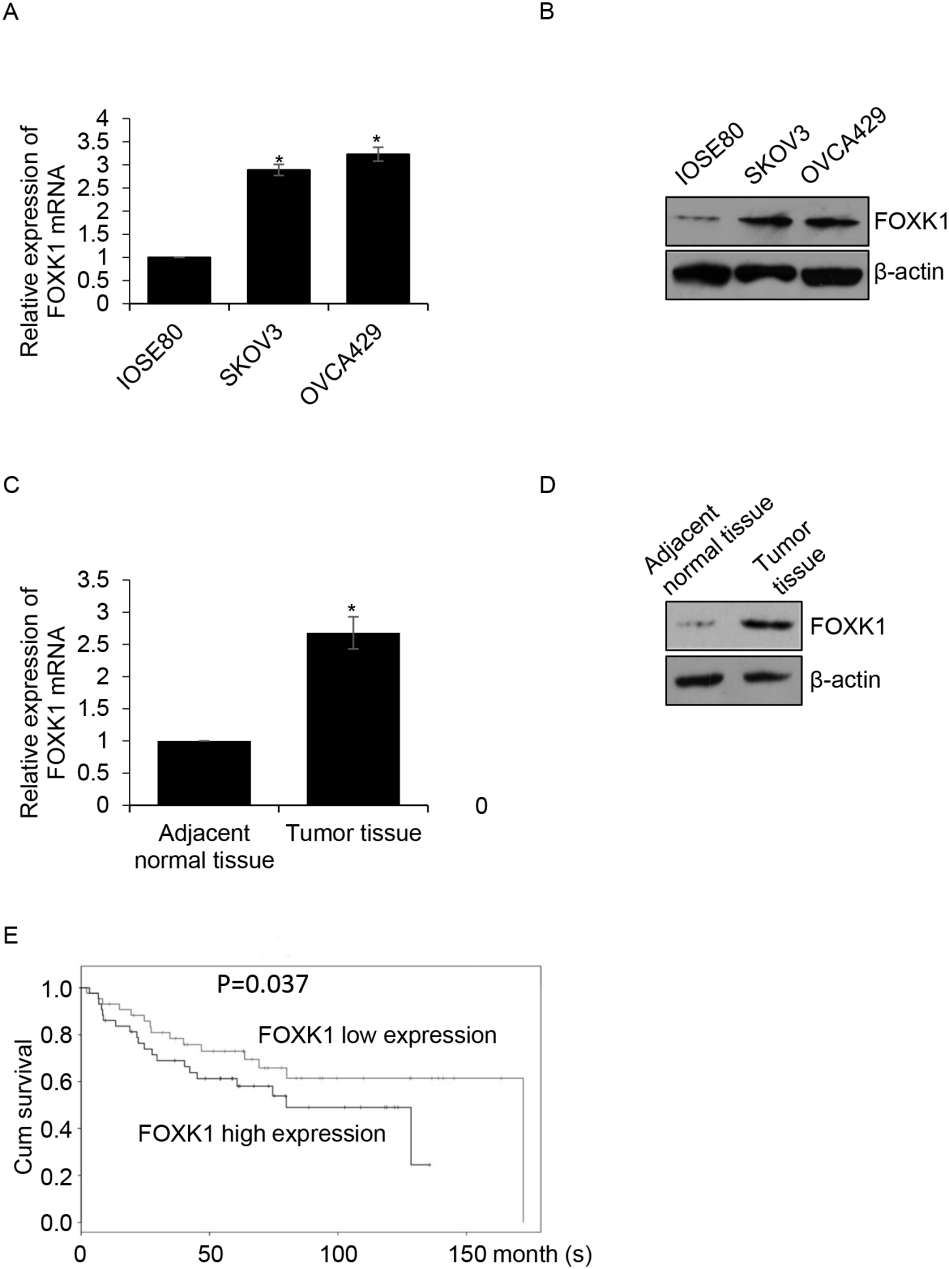
FOXK1 is up-regulated in ovarian cancer and the expression of FOXK1 is corrected with poor prognosis of ovarian cancer **(A)**. Total RNA was prepared from IOSE80, SKOV3 and OVCA429 cells, qRT-PCR was performed to detect the expression of FOXK1. **(B)**. Whole protein was prepared from IOSE80, SKOV3 and OVCA429 cells, western blotting was performed to detect the expression of FOXK1. **(C)**. Total RNA was prepared from 87 pairs’ tumor tissue samples and adjacent normal tissue samples, qRT-PCR was performed to detect the expression of FOXK1. **(D)**. Western blotting was used to detect the expression of FOXK1 in tumor tissue samples and adjacent normal tissue samples. **(E)**. The relationship between FOXK1 expression and survival curve was analyzed by Kaplan Meier method.

To analyze the function of FOXK1 in ovarian cancer, we first detected the expression of FOXK1 in different ovarian cancer cell lines, SKOV3 and OVCA429, human normal ovarian cell lines IOSE80 was used as a control group. The results of qRT-PCR and western blotting indicated FOXK1 was high expression in ovarian cancer cell lines, SKOV3 and OVCA429, compared with that in IOSE80 cells (Figure 1A and 1B). Subsequently, we collected 87 pairs’ ovarian cancer tissue samples and adjacent normal tissue samples from patients who were diagnosed as ovarian cancer in Affiliated Tumor Hospital of Guangxi Medical University during 2015-2016, then qRT-PCR and western blotting analysis were performed to determine the expression of FOXK1. Interestingly, we found that FOXK1 was up-regulated in ovarian cancer samples (Figure 1C and 1D). Moreover, we found high expression of FOXK1 was correlated with multiple clinic pathologic in ovarian cancer, including tumor size, pathological stage and metastasis. However, there were no obviously correlation with age and differentiation. Furthermore, we analyzed the relationship between FOXK1 and survival curve in ovarian cancer. The result suggested that the patients who had high expression of FOXK1 had shorter survival time than the patients who had low expression of FOXK1 (Figure 1E, P=0.037). In conclusion, we hypothesize that FOXK1 plays an important function in ovarian cancer.

### Overexpression of FOXK1 facilitates growth of ovarian cancer cells

**Figure 2:**
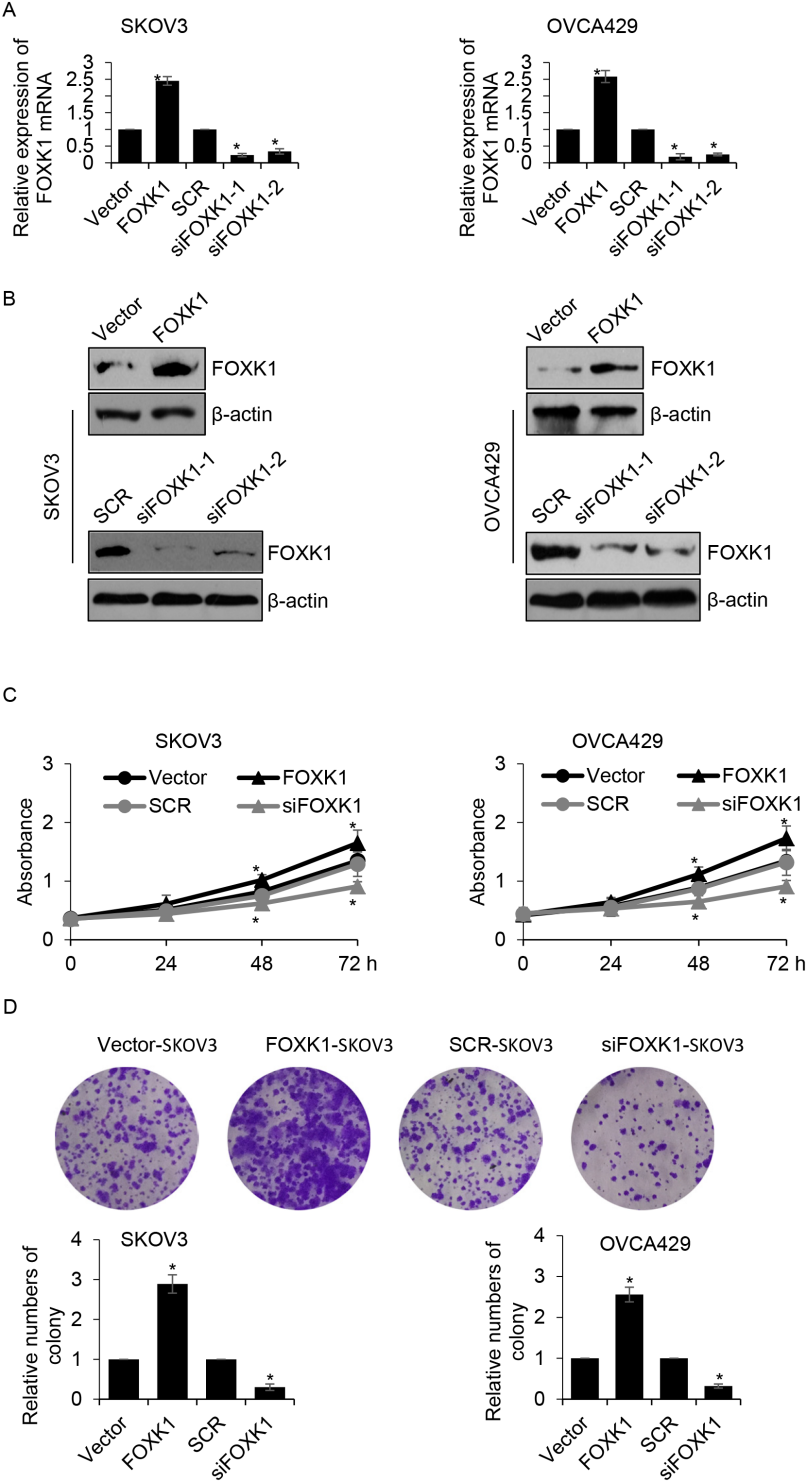
Overexpression of FOXK1 facilitates growth of ovarian caner cells **(A)**. SKOV3 and OVCA429 cells were transfected with empty plasmid (vector), FLAG-FOXK1, scramble siRNA (SCR), siFOXK1, respectively. The mRNA level of FOXK1 was determined by qRT-PCR. **(B)**. FOXK1 was overexpressed or silenced in SKOV3 and OVCA429 cells, respectively. The protein level of FOXK1 was determined by western blotting. **(C)**. FOXK1 was overexpressed or silenced in SKOV3 and OVCA429 cells, respectively. CCK-8 analysis was used to detect the effect of FOXK1 on cell proliferation. **(D)**. FOXK1 was overexpressed or silenced in SKOV3 and OVCA429 cells, respectively. Colony formation analysis was used to detect the effect of FOXK1 on cell proliferation.

In order to investigate the function of FOXK1 in ovarian cancer, we overexpressed or knocked down FOXK1 in SKOV3 or OVCA429 cells, respectively. Next, western blotting and qRT-PCR analysis were performed to determine the expression of FOXK1 in SKOV3 or OVCA429 cells. The results revealed that the expression of FOXK1 was obviously increased while cells were transfected with FLAG-FOXK1, compared with that of control group. In addition, the expression of FOXK1 was significantly decreased while cells were transfected with FOXK1 siRNA, compared with that of scramble siRNA (SCR) group. Moreover, we found siFOXK1-1 was more efficiency than siFOXK1-2, so siFOXK1-1 was used for the further experiments (Figure 2A and 2B). Because of the correlation between the expression of FOXK1 and tumor size, we assumed whether FOXK1 promoted ovarian cancer cells proliferation. Next, CCK-8 assay was used to detect the effect of FOXK1 on cell growth. The results suggested that SKOV3 cells transfected with FOXK1 grew faster than those transfected with vector (Figure 2C). On the contrary, the FOXK1-depleted cells grew slower than those transfected with SCR. The similar results were observed in OVCA429 cells (Figure 2C). Next, colony formation analysis was performed to determine the effect of FOXK1 on cell proliferation. The results showed that FOXK1 remarkably increased the number of colonies, on the contrary, FOXK1 inhibition decreased the number of colonies (Figure 2D). In conclusion, FOXK1 facilitates ovarian cancer cell proliferation.

### FOXK1 promotes G_1_/S phase transition, but has no effect on cell apoptosis

**Figure 3:**
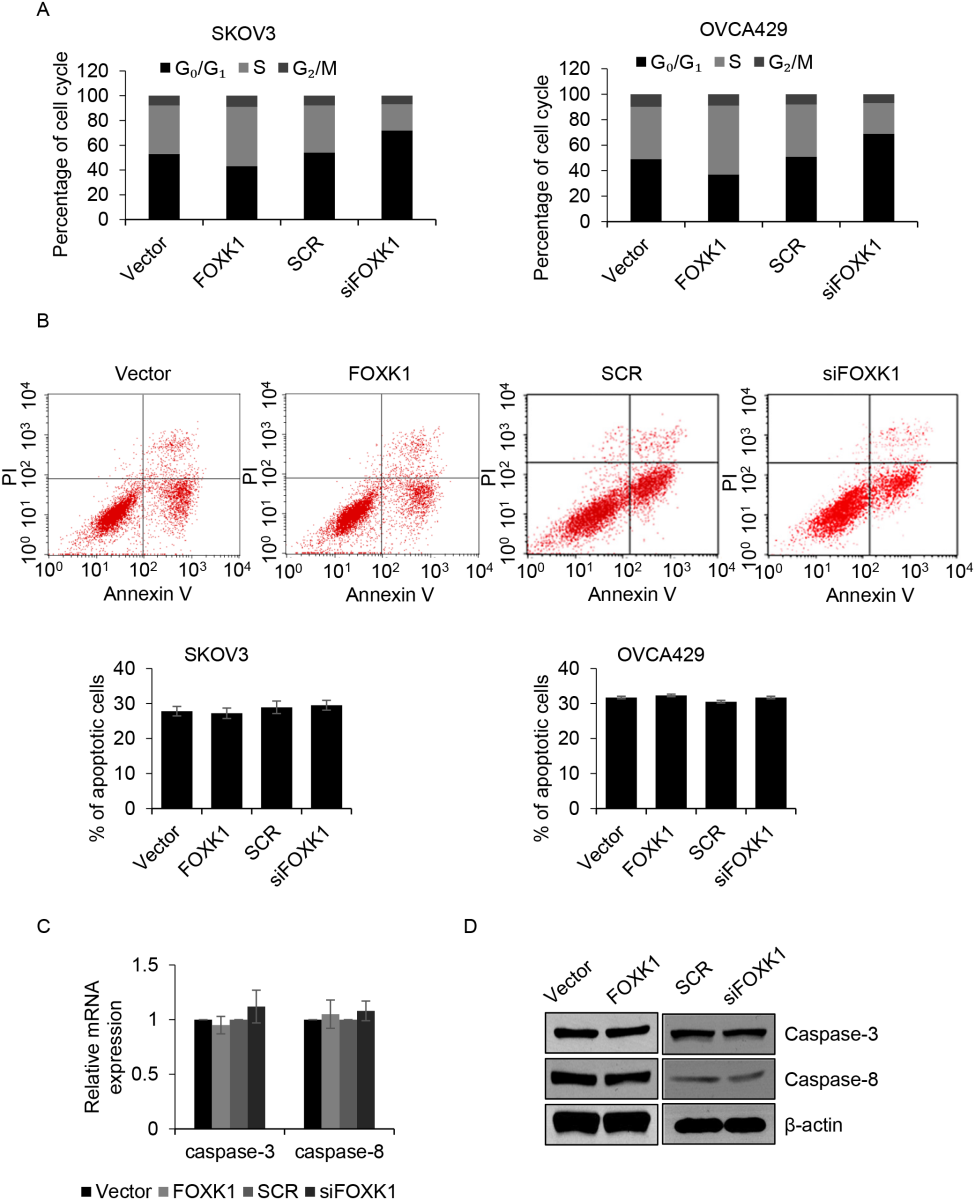
FOXK1 promotes G1/S transition, but has no effect on cell apoptosis **(A)**. SKOV3 and OVCA429 cells were transfected with empty plasmid (vector), FLAG-FOXK1, scramble siRNA (SCR), siFOXK1, respectively. Cell cycle was assessed by flow cytometer. **(B)**. FOXK1 was overexpressed or silenced in SKOV3 and OVCA429 cells, respectively. After incubation with CPT for 6 h, numbers of apoptotic cell was detected by flow cytometer. **(C)**. FOXK1 was overexpressed or silenced in SKOV3 cells, respectively. The mRNA level of caspase-3 and caspase-8 were detected by qRT-PCR. **(D)**. FOXK1 was overexpressed or silenced in SKOV3 cells, respectively. The protein level of caspase-3 and caspase-8 were detected by western blotting.

Subsequently, we further decipher the function of FOXK1 on cell cycle. Flow cytometry analysis indicated that overexpression of FOXK1 markedly improved the percentage of cells in the S phase, but the number of cells in G_0_/G_1_ phase was decreased. On the contrary, inhibition of FOXK1 strongly decreased the percentage of cells in S phase, but the number of cells in G_0_/G_1_ phase was increased (Figure 3A). Cell apoptosis also decrease cell proliferation, so we next detected if FOXK1 influenced cell apoptosis. The results demonstrated that FOXK1 had no effect on cell apoptosis (Figure 3B). Caspase-3 and caspase-8 as cell apoptosis markers, next, we detect whether they were activated or inhibited by FOXK1. As shown in Figure 3C and 3D, the mRNA and protein levels of caspase-3 and caspase-8 were not activated or inhibited by FOXK1. Together, FOXK1 facilitates cell proliferation through promoting G_1_/S phase transition.

### FOXK1 improves the migration and invasion ability of ovarian cancer cells

**Figure 4:**
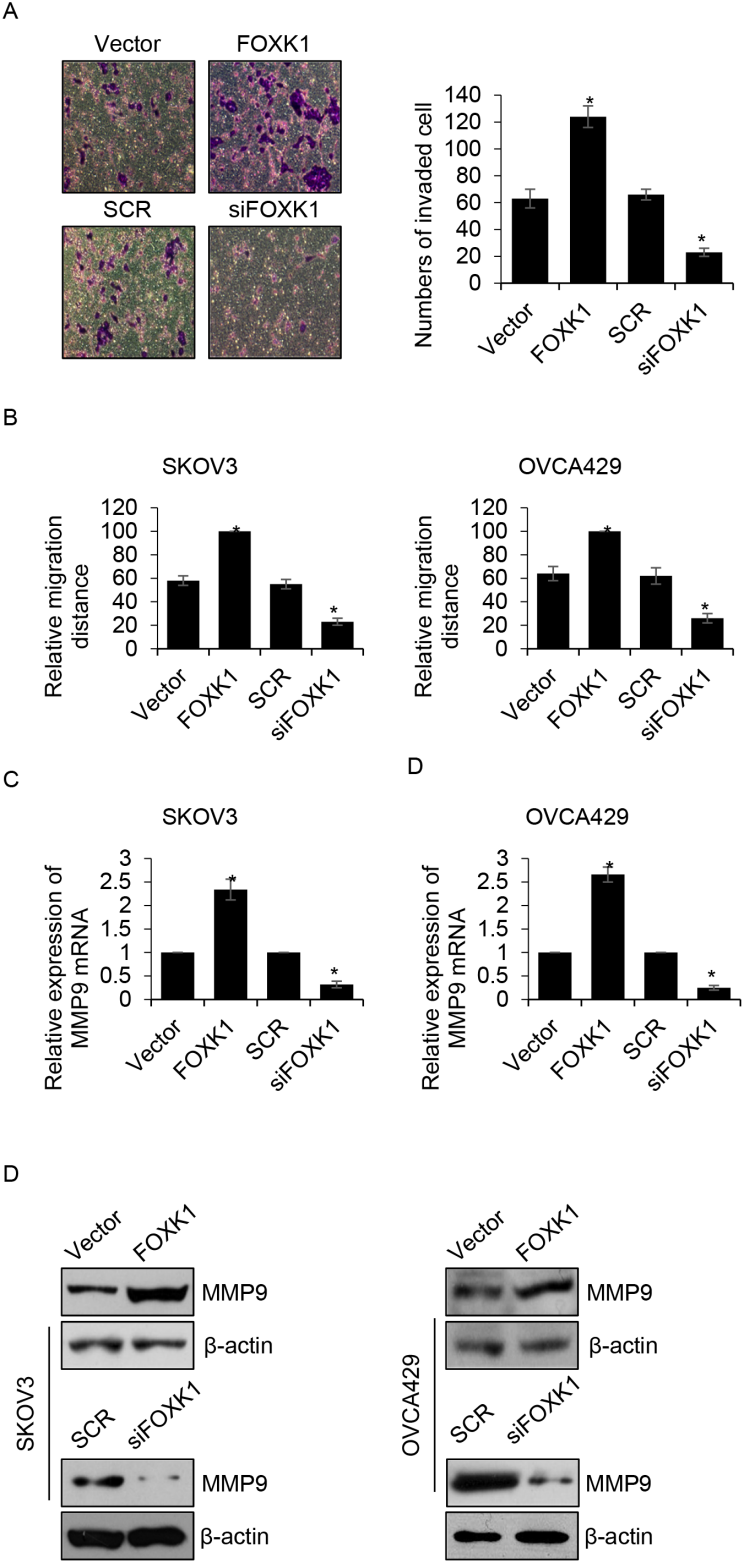
FOXK1 improves the migration and invasion ability of ovarian cancer cells **(A)**. OVCA429 cells were transfected with empty vector, FLAG-FOXK1, scramble siRNA (SCR), siFOXK1, respectively. Transwell invasion assay was used to investigate the effect of FOXK1 on cancer cell invasion. **(B)**. FOXK1 was overexpressed or silenced in SKOV3 and OVCA429 cells, respectively. After scratch by peptide, relative migration distance was measured at different time points. **(C)**. FOXK1 was overexpressed or silenced in SKOV3 and OVCA429 cells, respectively. The mRNA level of MMP-9 was detected by qRT-PCR. **(D)**. FOXK1 was overexpressed or silenced in SKOV3 and OVCA429 cells, respectively. The protein level of MMP-9 was detected by western blotting.

We found that the expression of FOXK1 was correlated with metastasis, so we hypothesized that FOXK1 incresed cancer cell metastasis. To verify our hypothesis, we first performed transwell analysis, the result indicated that ectopic expression of FOXK1 obviously promoted cell invasion, the mean invaded cells were 63 ± 7 and 124 ± 8 for vector-or FOXK1-transfected OVCA429 cells, respectively (Figure 4A). However, FOXK1 inhibition dramatically increased cell invasion, the mean relative migration distance were 66 ± 4 and 23 ± 3 for SCR-or siFOXK-transfected OVCA429 cells, respectively (Figure 4A). Subsequently, wound healing analysis was performed to detect the effect of FOXK1 on cell migration. We found ectopic expression of FOXK1 enhanced the migration ability of SKOV3 and OVCA429 cells, the mean relative migration distance were increased when FOXK1 was overexpressed in SKOV3 cells (Figure 4B). Whereas, FOXK1 inhibition suppressed the migration ability of SKOV3 cells (Figure 4B). The similar results were observed in OVCA429 cells (Figure 4B). MMP-9, a marker of cell invasion, has been found to increase tumor growth and metastasis in ovarian cancer [20-22]. We next detected whether FOXK1 increased MMP-9 expression. Both in SKOV3 and OVCA429 cells, the expression of MMP-9 were up-regulated by FOXK1 (Figure 4C and 4D). Meanwhile, the expression of MMP-9 was decreased when FOXK1 was knocked down (Figure 4C and 4D). In order to investigate whether FOXK1 transcriptionally increased MMP-9, we performed ChIP assay and luciferase reporter assay, the results indicated that FOXK1 didn’t transcriptionally increased MMP-9. FOXK1 might increase MMP-9 expression through regulation of any other proteins.

### FOXK1 transcriptionally inhibits *p21* in ovarian cancer cells

**Figure 5:**
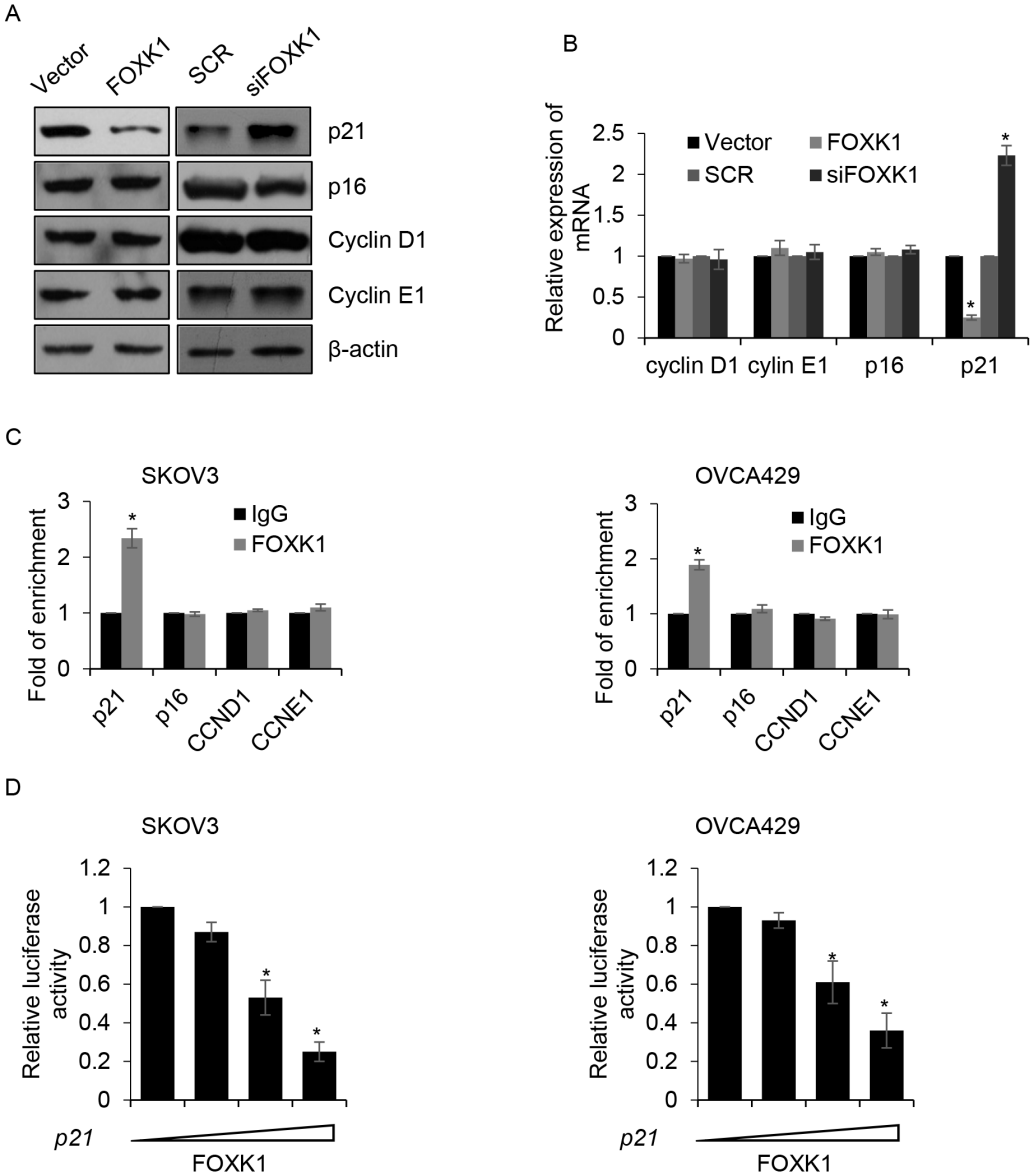
FOXK1 transcriptionally inhibits *p21* in ovarian cancer cells **(A)**. SKOV3 cells were transfected with empty plasmid (vector), FLAG-FOXK1, scramble siRNA (SCR), siFOXK1, respectively. The protein levels of cyclin D1, cyclin E1, p16 and p21 were detected by western blotting. **(B)**. FOXK1 was overexpressed or silenced in SKOV3 cells, respectively. The mRNA levels of cyclin D1, cyclin E1, p16 and p21 were detected by qRT-PCR. **(C)**. ChIP assay was performed to detect whether FOXK1 bound the promoter region of indicated gene in SKOV3 and OVCA429 cells. **(D)**. SKOV3 and OVCA429 cells were co-transfected with FOXK1, *Renilla*, pGL4-p21. 24 h after transfection, luciferase reporter assay was performed.

Cell cycle was regulated by numerous protein, including promoting cell cycle protein, such as cyclin D1 and cyclin E1, and inhibiting cell cycle protein, such as p16 and p21. To determine whether the effect of FOXK1 on G_1_/S phase transition through regulating the expression of these protein, we overexpressed or knocked down FOXK1 in SKOV3 cells, and performed Western blotting to detect the expression of those protein. The result revealed the expression of p21 was obviously suppressed when FOXK1 was overexpressed; however, FOXK1 inhibition promoted the expression of p21. But there were little effect on the expression of cyclin D1, cyclin E1 and p16 (Figure 5A). Furthermore, qRT-PCR suggested the mRNA level of p21 was also increased by FOXK1 (Figure 5B). Above results indicated that p21 might be transcriptionally increased by FOXK1. Subsequently, we performed ChIP analysis, the results showed FOXK1 interacted with the promotor region of *p21*, but not the promotor region of *CCND1, CCNE1* and *p16* (Figure 5C). Next, we sub-cloned the promotor region of *p16, p21, CCND1* and *CCNE1* into pGL4 plasmid. Luciferase reporter assay was performed to determine whether FOXK1 transcriptionally increased *p16*, *p21*, *CCND1* and *CCNE1*. Consisted with our previous work, we found only *p21* was transcriptionally inhibited by FOXK1 (Figure 5D), but FOXK1 had no effect on *p21*, *CCND1* and *CCNE1* (data not shown). In conclusion, FOXK1 promotes cell proliferation through transcriptionally inhibits *p21*.

## DISCUSSION

The members of FOX transcription factor family have different function during embryogenesis [23-25]. There are several reports indicate that FOX protein plays essential role in established cancers [9-11]. For instance, FOXA1 is high expression in multiple cancers, including pancreatic cancer [26], glioma cancer [27], breast cancer [28], prostate cancer [29] and bladder cancer [30]. Moreover, FOXK1 has been found to high express in multiple cancers, such as colorectal carcinoma [31].

In this study, we used ovarian tissue samples to explore FOXK1 expression profiles in adjacent normal tissues and cancer tissues. We found FOXK1 was high expression in cancer tissues, meanwhile, FOXK1 was up-regulated in ovarian cancer cell lines, so FOXK1 might act as an oncogene. In addition, survival curve analysis demonstrated a dramatically worse overall survival for ovarian cancer patients who had high expression of FOXK1, indicating that expression of FOXK1 was correlated with poor prognosis in ovarian cancer.

Recently, there were several researches revealed that FOXK1 facilitated proliferation, invasion and metastasis in different cancer [31-33]. Wang *et al.* found that FOXK1 and FOXK2 promote DVL translocating into nucleus, thereby regulation of the Wnt/*β*-catenin signaling [16]. However, the function of FOXK1 in ovarian cancer remains unknown. Consistently, our works have shown that FOXK1 promoted cell proliferation and invasion in ovarian cancer. These findings revealed that abnormal expression of FOXK1 might be an essential mechanism underlying cancer proliferation and metastasis.

FOXK1, a transcription factor, was identified as a crucial regulator for myogenic stem cell proliferation [12, 34, 35]. We identified that p21 as a downstream target of FOXK1, FOXK1 promoted G_1_/S phase transition through transcriptionally inhibiting *p21*. However, FOXK1 had no effect on cell apoptosis.

The detail mechanism of FOXK1 in metastasis remains unclear. EMT is a complex process which is widespread in malignant tumor. The main character of EMT is gain of mesenchymal character and loss of epithelial character, resulting epithelial cells transformed into mesenchymal cells. Recently, several members of the FOX family have been found to regulate EMT. FOXQ1 facilitated metastasis in colorectal carcinoma cells which had undergone TGF-*β*-induced EMT [36]. Moreover, FOXC2 was high expression in invasive ovarian cancer tissues and cell lines [37]. Furthermore, FOXC2 maintained TGF-*β*1-induced EMT. Recently report indicates that FOXK1 regulates EMT in gastric cancer (GC) [32]. So we assumed that FOXK1 might promote metastasis through regulation of EMT in ovarian cancer.

In conclusion, our work firstly find that FOXK1 is up-regulated in ovarian cancer tissues and cells. Moreover, we observe that FOXK1 has a relationship with poor prognosis. In addition, we find that FOXK1 promotes G_1_/S phase transition through transcriptionally inhibiting *p21.* Furthermore, FOXK1 facilitates invasion in ovarian cancer cell. Together, our study explain the function of FOXK1 in ovarian cancer and indicated that FOXK1 plays an essential function in mediating ovarian caner progression, and serves as a therapeutic target for ovarian cancer.

## MATERIALS AND METHODS

### Cell culture and ovarian tissue

Human ovarian cancer cell lines, SKOV3 and OVCA429 and human normal ovarian cell lines IOSE80 were purchased from the American Type Culture Collection (ATCC, Manassas, VA, USA). Cells were maintained in DMEM (HyClone, Logan, UT, USA) supplemented with 1% Penicillin-Streptomycin solution (HyClone, Logan, UT, USA) and 10% FBS (HyClone, Logan, UT, USA) at 37°C with 5% CO_2_. For ovarian tissue sample experiments, the patients had known our experiments before we collected their tissue samples. Our tissue sample experiments were approved by Ethics Committee of the Affiliated Tumor Hospital of Guangxi Medical University.

### Cell transfection

Cells were transfected with scramble siRNA (SCR) or FOXK1 siRNA (siFOXK1) at density of 30%-40% by Lipofectamine RNAiMax reagent (Thermo Fisher Scientific, Waltham, MA, USA). 48 h after transfection, cells were collected and used for the further experiments.

### Western blotting

Cell lysate was prepared by RIPA lysis buffer (Beyotime, Jiangsu, People’s Republic of China) supplemented with cocktail, a protease inhibitor (Thermo Fisher Scientific, Waltham, MA, USA). Equal amount of proteins (45 μg) were separated on 10% SDS-PAGE, then proteins were transferred onto nitrocellulose filter membranes. 5% non-fat milk solution was used to block nonspecific antigen on the membranes, and the membranes were washed by PBST solution for three times. Subsequently, specific antibodies were used to incubate membranes at 4°C overnight. Next, the membranes were washed with PBST for three times and incubated with second antibody (1:5000; Sigma, USA) at room temperature for 1 h. The blot of specific protein was visualized by ECL (Millipore, Bedford, MA, USA).

### qRT-PCR

Total RNA was prepared from cells by TRIzol reagent (Invitrogen, Carlsbad, CA, USA), and then RNA were reverse transcribed into cDNA by PrimeScript^®^ 1st strand cDNA synthesis kit (TaKaRa, Tokyo, Japan). SYBR® Green Real-time PCR Master Mix (Sigma, USA) was used to determine the mRNA level of indicated gene by 7900HT qRT-PCR system. GAPDH was served as an internal control. Relative mRNA levels of indicated gene were analyzed using the 2^-ΔΔCt^method.

### Cell cycle assay

Cell cycle assay was used to detect whether FOXK1 regulated cell cycle. In brief, FOXK1 was overexpressed or silenced in SKOV3 and OVCA429 cells, next, cells were collected, follow by fixed with ice-cold 70% ethanol for 20 min and stained with propidium iodide (PI, Transgene, Beijing, People’s Republic of China) at room temperature for 20 min. Cell cycle was determined through flow cytometry (FACS Calibur, BD, San Diego, CA, USA).

### Cell apoptosis assay

Cell apoptosis assay was performed to assess the effect of FOXK1 on cell apoptosis. Briefly, FOXK1 was overexpressed or silenced in SKOV3 and OVCA429 cells, subsequently, cells were collected and resuspended in 100 μl binding buffer. Next, 5 μl annexin V-FITC and 5 μl PI (Jingmei Biotech, Shenzhen, People’s Republic of China) were added in cell solution, the cells were incubated at room temperature for 15 min and then cell apoptosis was analyzed by flow cytometry (FACS Calibur, BD, San Diego, CA, USA).

### Wound healing assay

Wound healing assay was performed to assess the effect of FOXK1 on cell migration. Briefly, SKOV3 and OVCA429 cells were transfected with vector, FOXK1, SCR, FOXK1 siRNA, respecticly. When the cells density reached 90%-100%, a 20 μl pipette was used to create a scratch. Meanwhile, cells were incubated with serum-free DMEM. Scratches were observed under a microscope. The distance of scratch was measured at different time points (0 h and 24 h) by Image Pro-Plus 6.0 software (Media Cybernetics, USA).

### Transwell invasion assay

Transwell invasion assay was performed to assess the effect of FOXK1 on cell invasion. Transwell chamber (8 μm pore size, Corning Costar, Corning, NY, USA) was used in this experiments. After transfection, 10,000 cells were resuspended in serum-free DMEM and placed into the upper well which coated with 100 μl Matrigel (BD, San Diego, CA, USA). Simultaneously, the lower well of the chamber was filled with DMEM containing 10% FBS. Cells were maintained at 37°C for 24 h. Subsequently, cells on the upper surface of the chambers were removed by cotton swab, the cells on the lower surface of the chambers were stained with 0.1% crystal violet and counted under a microscope.

### CCK-8 analysis

CCK-8 analysis was used to determine the function of FOXK1 on cell proliferation. SKOV3 and OVCA429 cells were transfected with vector, FOXK1, SCR, siFOXK1 using Lipo 2000 reagent (Thermo Fisher Scientific, Waltham, MA, USA), respectively. 48 h after transfection, approximately 4,000 cells were placed into each well of 96-well plates. CCK-8 solution was added at 0 h, 24 h, 48 h and 72 h after placing. After adding 10 μl CCK-8 solution, cells were incubated for 1 h at 37 °C. The absorbance was measured at 450 nm.

### Statistical analysis

The data were analyzed by Statistical Product and Service Solutions 19.0 (SPSS, IBM, USA). The comparison between two group was analyzed by Student’s *t*-test. The data was represented as mean ± SD. The *p* < 0.05 was considered as statistical significant (*p < 0.05).
